# Supplementary material for: Genome-Wide Association Study Identified Novel SNPs Associated with Chlorophyll Content in Maize
Source: Genes (Basel). 2023 Apr 29;14(5):1010. doi: 10.3390/genes14051010 (PMC10218100; doi:10.3390/genes14051010)
Supplement: Supplementary file 1 [file genes-14-01010-s001.zip › genes-2335884-supplementary.pdf]

Table S1. Phenotypic description of chlorophyll content in the maize association population

Site[a]	Means±SD（%）	Range (%)
17FS	54.81±4.57	36.23-68.85
17LD	49.63±7.89	27.23-62.66
BLUP	51.96±3.68	44.78-58.61

[a] 17FS, 2017 Fushun; 17LD, 2017 Ledong; BLUP, the Best Unbiased Linear Predictive value of 17FS and 17LD

Supplementary Table S2. Single-nucleotide polymorphism (SNP) chromosomal positions and candidate genes significantly associated with the chlorophyll content and chlorophyll content dynamic changes identified by genome-wide association study.

Trait	SNP	Chr	Position (bp)	P-value	Gene	Gene interval (bp)	Annotation	Pathway
17BLUP chlorophyll content	2435653-60-G	5	72634276	6.04E-06	Zm00001d015018	(Chr5: 72612562..72615969)	Unknown	Unknown
					Zm00001d015021	(Chr5: 72640943..72645739)	Unknown	Unknown
					Zm00001d015019	(Chr5: 72632612..72635911)	Unknown	Unknown
					Zm00001d015020	(Chr5: 72635763..72642535)	Unknown	Unknown
					Zm00001d015024	(Chr5: 72668409..72678234)	Unknown	Unknown
					Zm00001d015022	(Chr5: 72642370..72665247)	Unknown	Unknown
					Zm00001d015023	(Chr5: 72666251..72669943)	Unknown	Unknown
	Marker. 247949	2	2.2E+08	9.10E-06	Zm00001d007009	(Chr2: 220125041..220148355)	DNAJ heat shock N-terminal domain-containing protein	Chloroplast targeting, photosystem II repair
					Zm00001d007011	(Chr2: 220152033..220155206)	ATP synthase	
					Zm00001d007010	(Chr2: 220151965..220155369)	Unknown	Unknown
					Zm00001d007012	(Chr2: 220170458..220175539)	CHLOROPLAST RNA-BINDING PROTEIN	
					Zm00001d007014	(Chr2: 220185401..220189333)	Unknown	Unknown
					Zm00001d007015	(Chr2: 220201942..220205547)	Unknown	Unknown
					Zm00001d007016	(Chr2: 220204271..220219191)	disease resistance protein RGA2	Disease-resistant
					Zm00001d007017	(Chr2: 220217348..220221222)	Thioredoxin-like protein AAED1 chloroplastic	Electronic circulation and daylighting
					Zm00001d007018	(Chr2: 220219528..220223120)	Unknown	Unknown

17FS chlorophyll content	2410636-41-G	1	85173718	9.14E-07	Zm00001d029747	(Chr1: 85171204..85175964)	peroxidase	Redox
					Zm00001d029746	(Chr1: 85161728..85170290)	Unknown	Unknown
	2376873-7-G	10	1.48E+08	7.08E-06	Zm00001d026565	(Chr10:148158667..148161739)	Unknown	Unknown
					Zm00001d026564	(Chr10:148157881..148160953)	Unknown	Unknown
					Zm00001d026563	(Chr10:148153479..148157015)	AP2/EREBP transcription factor 40	Plant growth and development
					Zm00001d026567	(Chr10: 148174426..148180373)	Unknown	Unknown
					Zm00001d026569	(Chr10:148185247..148190302)	chloroplastic palmitoyl-acyl carrier protein thioesterase	De novo synthesis of fatty acids
					Zm00001d026568	(Chr10: 148184610..148195615)	pentatricopeptide repeat-containing protein	Chloroplast development
					Zm00001d026572	(Chr10: 148199415..148204255)	Unknown	Unknown
					Zm00001d026571	(Chr10: 148193776..148200881)	Unknown	Unknown
					Zm00001d026573	(Chr10: 148201459..148207853)	5-methylthioribose kinase	Methylthioadenosine (MTA) cycle
					Zm00001d026574	(Chr10:148208912..148217798)	UDP-D-galacturonate	Homogalacturonan biosynthesis
					Zm00001d026570	(Chr10:148192633..148197349)	Unknown	Unknown
					Zm00001d026566	(Chr10:148162162..148174843)	Unknown	Unknown
	2439854-28-A	7	1.44E+08	9.45E-06	Zm00001d021162	(Chr7: 144349898..144354922)	polymerase delta 4	DNA replication
17LD chlorophyll content	Marker.114209	10	2743583	7.60E-08	Zm00001d023311	(Chr10: 2741866..2755857)	Unknown	Unknown
					Zm00001d023312	(Chr10: 2755975..2760003)	transmembrane protein, putative (DUF1191)	Transmembrane
					Zm00001d023313	(Chr10: 2767649..2773867)	Phototropic-responsive NPH3 family protein	

				Zm00001d023314	(Chr10: 2773581..2778902)	ROOT PHOTOTROPISM 2, RPT2	Early signaling component in the phototrophic
				Zm00001d023310	(Chr10: 2715895..2719365)	Unknown	Unknown
Marker. 127361	10	44716397	1.02E-06				
Marker. 252961	2	2.33E+08	1.17E-06	Zm00001d007477	(Chr2: 232636792..232641852)	wall-associated receptor kinase	Cell wall composition
				Zm00001d007479	(Chr2: 232669019..232675794)	(bsdtf1 - BSD-transcription factor 1)	Transcription factor
				Zm00001d007478	(Chr2: 232668331..232671687)	phytosulfokines	Plant growth and development
				Zm00001d007480	(Chr2: 232676089..232679162)	Unknown	Unknown
				Zm00001d007481	(Chr2: 232694058..232698314)	ornithine decarboxylase	superpathway of polyamine biosynthesis，putrescine biosynthesis
				Zm00001d007482	(Chr2: 232699572..232709088)	Unknown	Unknown
2434834 -58-G	1	2.96E+08	3.47E-06	Zm00001d034528	(Chr1: 295705725..295711971)	ubiquinol-cytochrome c reductase complex protein	Electron transport
				Zm00001d034529	(Chr1: 295711501..295717880)	Unknown	Unknown
				Zm00001d034530	(Chr1: 295744732..295748445)	Unknown	Unknown
				Zm00001d034532	(Chr1: 295766368..295773997)	SNF7 family protein	Autophagic degradation
				Zm00001d034531	(Chr1: 295749032..295761989)	lipoate acetyltransferase	Apyruvate decarboxylation to acetyl CoA
				Zm00001d023022	(Chr1: 295783194..295791255)	Unknown	Unknown
				Zm00001d034533	(Chr1: 295777054..295784040)	tRNA-splicing ligase (DUF239)	Transcriptional translation
				Zm00001d034534	(Chr1: 295788307..295792693)	sterol O-acyltransferase, putative (DUF1639)	Composition of fatty acyl esters of Phytosterols

				Zm00001d034535	(Chr1: 295805133..295809336)	Unknown	Unknown
Marker. 76989	1	2.15E+08	6.33E-06				
2456265 -10-T	1	2.95E+08	6.92E-06	Zm00001d034511	(Chr1: 295099025..295106866)	Hydroxyproline O-galactosyltransferase GALT4	Plant growth and development
Marker. 190636	2	43281907	6.97E-06	Zm00001d000804	(Chr2: 43258554..43269536)	Unknown	Unknown
				Zm00001d003403	(Chr2: 43279990..43285413)	(aaap10 - amino acid/auxin permease10)	Amino acid transportation
				Zm00001d003402	(Chr2: 43265762..43271211)	Unknown	Unknown
				Zm00001d003404	(Chr2: 43284025..43288746)	transmembrane protein	Transmembrane
				Zm00001d003405	(Chr2: 43285551..43289511)	protease inhibitor/seed storage/LTP family protein	
				Zm00001d003406	(Chr2: 43298285..43304459)	actin binding protein	Plant growth and development
Marker. 404379	4	1.86E+08	8.30E-06	Zm00001d052276	(Chr4: 185634679..185646700)	RNA-processing, Lsm domain-containing protein	RNA silencing
				Zm00001d052277	(Chr4: 185650634..185655330)	PADRE protein.	Disease-resistant
				Zm00001d052278	(Chr4: 185659171..185669321)	xylose isomerase	Xylose degradation
				Zm00001d026955	(Chr4: 185681571..185685071)	Unknown	Unknown
				Zm00001d052275	(Chr4: 185619211..185622456)	Unknown	Unknown
Marker. 346487	4	8724268	8.32E-06	Zm00001d027597	(Chr1: 8674405..8678346)	Unknown	Unknown
				Zm00001d027598	(Chr1: 8676135..8681000)	cct101 - CO CO-LIKE TIMING OF CAB1 protein domain101)	Transcription factors, floral completion
				Zm00001d027599	(Chr1: 8699800..8704356)	alkane hydroxylase MAH1	Cuticular wax biosynthesis
				Zm00001d027600	(Chr1: 8702407..8705625)	Unknown	Unknown

					Zm00001d027601	(Chr1: 8772748..8777355)	behenate ω-hydroxylase	Suberin monomers biosynthesis
	2504165-22-G	5	2775822	9.93E-06	Zm00001d012982	(Chr5: 2770681..2776100)	NAD(P)-binding domain containing protein	
17LD chlorophyll content dynamic changes	2480050-10-T	5	33393858	7.24E-07	Zm00001d014126	(Chr5: 33392233..33396960)	UDP-glycosyltransferase	Cytokinin-O-glucosides biosynthesis
					Zm00001d014127	(Chr5: 33403923..33407737)	Unknown	Unknown
					Zm00001d014128	(Chr5: 33411909..33417021)	Unknown	Unknown
	Marker.571424	6	1.73E+08	7.40E-06	Zm00001d039222	(Chr6: 173160699..173165510)	cct40 - CO CO-LIKE TIMING OF CAB1 protein domain40)	Transcription factors, floral completion
					Zm00001d039221	(Chr6: 173158717..173163744)	nucleoside diphosphate kinase 4	
17FS chlorophyll content dynamic changes	2376873-7-G	10	1.48E+08	5.82E-06	Zm00001d026565	(Chr10: 148158667..148161739)	Unknown	Unknown
					Zm00001d026564	(Chr10: 148157881..148160953)	Unknown	Unknown
					Zm00001d026567	(Chr10: 148174426..148180373)	Unknown	Unknown
					Zm00001d026563	(Chr10: 148153479..148157015)	(ereb40 - AP2-EREBP-transcription factor 40)	Plant growth and development
					Zm00001d026568	(Chr10: 148184610..148195615)	pentatricopeptide repeat-containing protein, mitochondrial-like	Chloroplast development
					Zm00001d026569	(Chr10: 148185247..148190302)	palmitoyl-acyl carrier protein thioesterase, chloroplastic	De novo synthesis of fatty acids
					Zm00001d026572	(Chr10: 148199415..148204255)	Unknown	Unknown
					Zm00001d026571	(Chr10: 148193776..148200881)	Unknown	Unknown
					Zm00001d026573	(Chr10: 148201459..148207853)	5-methylthioribose kinase	Methylthioadenosine (MTA) cycle
					Zm00001d026574	(Chr10: 148208912..148217798)	UDP-D-galacturonate	Homogalacturonan biosynthesis

				Zm00001d026570	(Chr10: 148192633..148197349)	Unknown	Unknown
				Zm00001d026566	(Chr10: 148162162..148174843)	Unknown	Unknown
Marker. 407039	4	1.92E+08	6.74E-06	Zm00001d052523	(Chr4: 192070209..192077428)	Protein kinase superfamily protein	Positive phototropism and gravitropism.
				Zm00001d052525	(Chr4: 192079383..192086467)	putative calmodulin-binding family protein	Calmodulin-binding motif family protein

Table S3. Expression of different candidate genes in seven different tissues

gene	Embryo	Endosperm	Internode	seed	Anthers	Silks	Leaf
Zm00001d007009	8.1	7.1	7.9	18.1	5.1	9.7	4.6
Zm00001d007011	2568.7	2179	535.5	1608.8	951.7	1588.5	478.5
Zm00001d007012	15.1	27.5	9.9	3.4	6	3.5	4.7
Zm00001d007016	0.1	0	0.7	0.3	0.1	0.2	0.7
Zm00001d007017	0	0	0	0	0	0	0
Zm00001d029747	1	0.1	3.3	1.6	1.8	4	1.7
Zm00001d026563	0.5	0.1	0.2	0	0	0	2.3
Zm00001d026569	1.6	0.5	0	0.6	0.6	0.2	0.4
Zm00001d026568	1.7	0.5	0.4	0.8	0.7	0.5	0.3
Zm00001d026573	27.4	1.7	61.4	32.1	12.4	40.7	16.2
Zm00001d026574	0.1	0.2	0	0.4	0.6	0.3	0
Zm00001d021162	9.8	2.2	7.2	6.8	5.5	9	4.3
Zm00001d023312	7.7	1.9	0	0.2	0	0.1	0.1
Zm00001d023313	6.7	1	0.5	0.7	2.7	0.7	1.5
Zm00001d023314	0.1	0	3.6	0.1	7.9	7.6	80.5
Zm00001d007477	1.3	0.3	2.2	0.8	1.8	1.1	0.2
Zm00001d007479	7.5	1	3.2	4.3	2.2	2.7	1.1
Zm00001d007478	1.3	0.7	1.6	0	0	0.8	0.4
Zm00001d007481	0	0	0	0	3	0	0
Zm00001d034528	75.2	62.8	61.2	44.9	56	44.4	37.8
Zm00001d034532	3.6	13.8	15.4	19.8	15.4	10.5	17.5
Zm00001d034531	14.7	8.2	15.5	19.9	24.5	31.5	5.1
Zm00001d034533	23.6	5.8	2.4	5.9	4	11.1	3.6
Zm00001d034534	4.8	4.4	6.3	8.3	4.8	9.2	15.2
Zm00001d034511	1	2.8	1.9	2.4	7.4	0.4	0.5
Zm00001d003403	0	0	1.3	0	0.6	0	0
Zm00001d003404	0.2	0	1.3	0.5	5.7	5	18.2
Zm00001d003405	0	2.00E-01	0	0.1	0	0	0.1
Zm00001d003406	1.4	0.1	0.1	1.1	0	0.1	0
Zm00001d052276	16.1	9.1	10.3	16.2	7.9	11.4	5.7
Zm00001d052277	0.9	0.1	9.3	7.9	1.8	2.4	5.4
Zm00001d052278	0.2	0.1	0	0.7	0.4	0.4	0.1
Zm00001d027598	0	0	0	0.1	5.9	0	1
Zm00001d027599	0.1	0.3	0.9	13.8	1.1	2.4	0
Zm00001d027601	0	0	2.4	0.1	3.1	0.2	4.3
Zm00001d012982	1.1	0.1	7.7	12.8	4.1	6.3	26.1
Zm00001d012983	0	0	0.5	0.1	0.1	0	0
Zm00001d014126	0.1	0	2.8	0.3	1.1	0.9	16.1
Zm00001d039222	2.6	2.4	3.2	5.8	1.6	3.5	1.7
Zm00001d039221	20	19.8	27.4	12.5	13.3	13.2	3.5
Zm00001d052523	2.5	0.8	0.8	0.2	0.1	0	2.4

Table S4. Expression of candidate genes in leaves at 0, 6, 12, 18, 24 and 30 days after pollinating

Gene	0_DAP	6_DAP	12_DAP	18_DAP	24_DAP	30_DAP
Zm00001d007009	0.869791	0.954662	0.874111	0.987931	0.901623	0.862941
Zm00001d007011	2.560071	2.611473	2.796484	2.709799	2.878477	2.691364
Zm00001d007012	1.275274	1.02612	2.251757	1.738233	2.064008	1.194428
Zm00001d007016	0.428054	0.362639	0.364965	0.389694	0.34684	0.486913
Zm00001d007017	0	0	0	0	0	0
Zm00001d029747	0.704051	0.464642	0.420616	0.318076	0.36411	0.396889
Zm00001d026563	0.979483	1.225901	0.340563	0.309025	0.791024	0.983642
Zm00001d026569	0.09951	0	0.016674	0.008415	0.031328	0.094901
Zm00001d026568	0.197928	0.15857	0.332263	0.34485	0.288546	0.238334
Zm00001d026573	1.661228	1.666179	1.887768	1.68533	1.831661	1.684625
Zm00001d026574	0	0	0	0	0	0
Zm00001d021162	0.829456	1.003826	0.948375	1.044881	1.065509	1.002344
Zm00001d023312	0	0.122918	0	0	0	0
Zm00001d023313	0.406869	0.342442	0.227415	0.371327	0.150846	0.204083
Zm00001d023314	2.437529	2.613405	2.476507	2.535318	2.360184	2.486283
Zm00001d007477	0.127586	0.236793	0.148638	0.242085	0.260091	0.386022
Zm00001d007479	0.481043	0.785405	0.528968	0.519186	0.711913	0.724411
Zm00001d007478	0.379712	0	0	0	0.239819	0.155354
Zm00001d007481	0	0	0	0	0	0
Zm00001d034528	1.838083	1.867142	1.863886	1.875946	2.022193	1.868008
Zm00001d034532	1.201282	1.293811	1.034369	1.144331	1.20463	1.171009
Zm00001d034531	1.071834	1.159946	1.059522	1.120541	1.180636	1.084169
Zm00001d034533	0.420251	0.474891	0.569028	0.520696	0.174637	0.226862
Zm00001d034534	1.161302	1.342695	1.182221	1.145119	1.181546	1.060902
Zm00001d034511	0.353341	0.344932	0.335498	0.477496	0.358068	0.500446
Zm00001d003403	0.221312	0.319056	0	0	0	0
Zm00001d003404	1.667707	1.320999	1.788239	1.566468	1.605397	1.49729
Zm00001d003405	0	3.24E-09	0.372809	0.137727	0.282007	0.177736
Zm00001d003406	0.086866	0	0	0	0.027375	0.029106
Zm00001d052276	1.11234	1.283306	1.13266	1.305491	1.296347	1.277199
Zm00001d052277	0.64186	0.648453	0.540919	0.688467	0.784265	0.757555
Zm00001d052278	0	0	0	0	0	0
Zm00001d027598	0.632006	0.265807	0.52268	0.508538	0.399753	0.216771
Zm00001d027599	0.103756	0	0	0	0	0.244137
Zm00001d027601	0.667727	1.05589	1.482835	1.757981	1.501137	1.917897
Zm00001d012982	1.849065	1.799443	1.781306	1.719826	1.954301	1.859462
Zm00001d012983	0	0	0.018044	0.076018	0.120492	0
Zm00001d014126	1.419465	1.396007	1.468618	1.487162	1.531658	1.418281
Zm00001d039222	0.398737	0.582248	0.433065	0.392957	0.340805	0.375327
Zm00001d039221	0.930823	0.987484	0.917366	0.93787	1.046581	1.07497
Zm00001d052523	0.500793	0.543734	0.360057	0.443377	0.484126	0.684997

Zm00001d052525	1.471102	1.48713	2.018958	1.980788	1.83006	1.538987

Table S5. Chlorophyll content in 6 different varieties

ID	Chlorophyll content				
Liao7980	45.7	46.1	49.1	47.2	49.2
A801	44.6	43.7	46	47.4	44.4
29MIBZ2	43.6	41.6	42.8	44.3	43.5
PHVA9	41.8	40.4	32.6	40.7	38.5
LX9311	33.8	33.4	33.2	32.5	34.9
Dan330	36.3	36.2	34.1	36.1	35.9

Table S6. Primers used in this study

ZmTubulin1-F: GTGTCCTGTCCACCCACTCTCT

ZmTubulin1-R: GGAACTCGTTCACATCAACGTTC

Zm00001d026568F: ACAGAACGGGTGGTACGAAGA

Zm00001d026568R: AGTCCCAACACACACATTCCC

Zm00001d026569F: AACAAGCACGTGGGTGATGA

Zm00001d026569R: TAAGGTCGGAGTCGACGTACAT

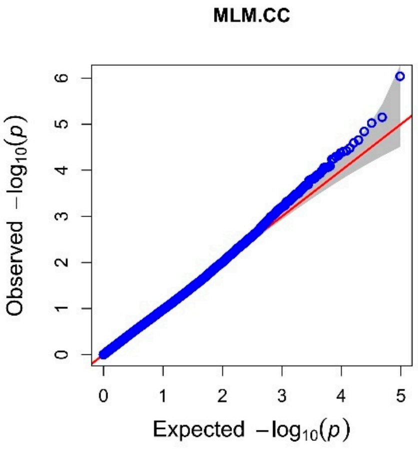

A

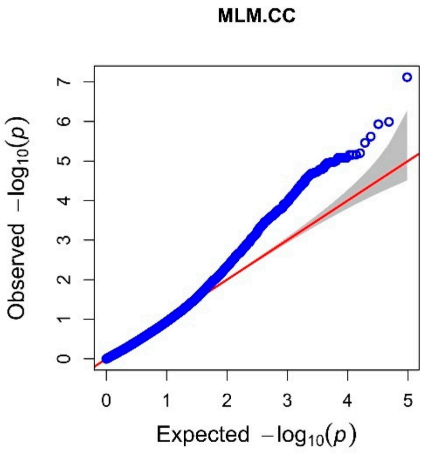

B

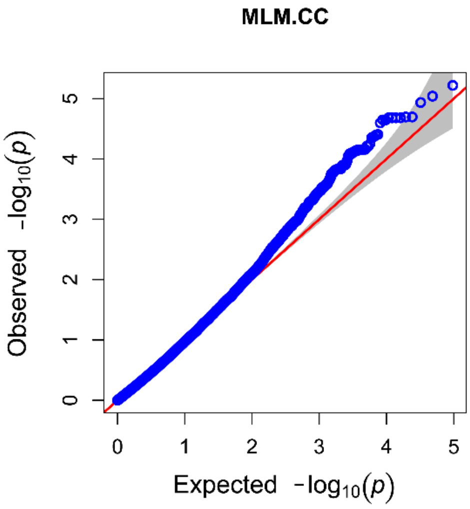

C

Figure S1. Quantile quantile-plot for chlorophyll content of maize ear leaf. The horizontal axis shows −log10 transformed expected P values, and the vertical axis indicates −log10 transformed observed P values. (A) 17FS; (B) 17LD; (C) BLUP.

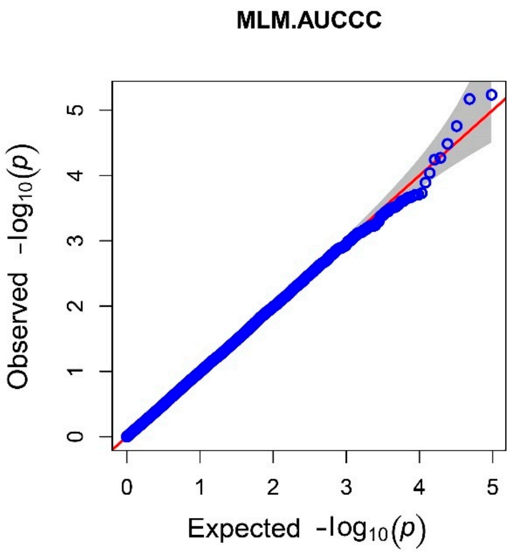

A

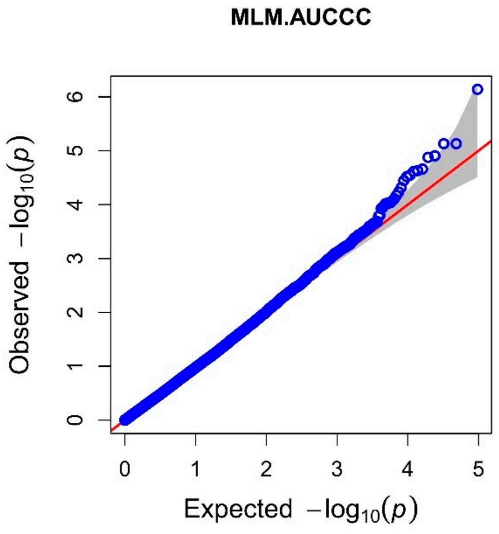

B

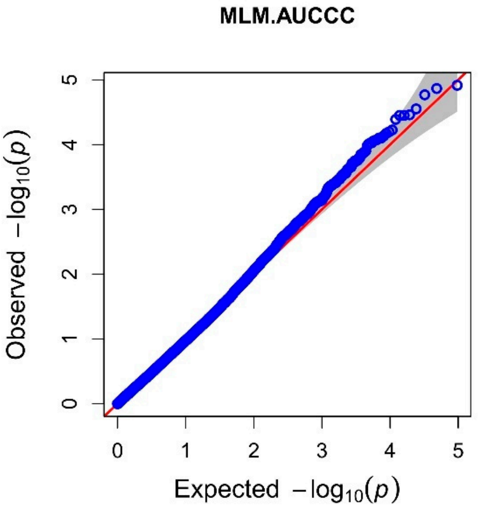

C

Figure S2. Quantile quantile-plot for chlorophyll content dynamic change of maize ear leaf. The horizontal axis shows −log10 transformed expected P values, and the vertical axis indicates −log10 transformed observed P values. (A) 17FS; (B) 17LD; (C) BLUP.
